# Cost of inappropriate prescriptions for uncomplicated malaria in Ghana

**DOI:** 10.1186/s12936-023-04581-8

**Published:** 2023-05-18

**Authors:** Genevieve Cecilia Aryeetey, Justice Nonvignon, Keziah Malm, Richmond Owusu, Bright Sasu Baabu, Nana Yaw Peprah, Samuel Agyei Agyemang, Jacob Novignon, Samuel Amon, Duah Dwomoh, Moses Aikins

**Affiliations:** 1 Department of Health Policy, Planning and Management, School of Public Health, University of Ghana, Legon, Accra, Ghana; 2 National Malaria Elimination Programme, Ghana Health Service, Accra, Ghana; 3 Department of Economics, Kwame Nkrumah University of Science and Technology, Kumasi, Ghana; 4 Department of Biostatistics, School of Public Health, University of Ghana, Legon, Accra, Ghana

**Keywords:** Cost, Ghana, Inappropriate, Malaria, Prescription

## Abstract

**Background:**

Malaria remains a common course of morbidity in many sub-Saharan African countries. While treatment options have improved in recent times, inappropriate prescription seems conventional among providers, increasing the burden on patients and society. This study examined the cost of inappropriate prescriptions for uncomplicated malaria treatment in Ghana.

**Methods:**

This study used retrospective data collected from January to December 2016 in 27 selected facilities, under different ownership in three regions of the country, mainly Volta, Upper East and Brong Ahafo. Stratified random sampling technique was used to extract 1625 outpatient folders of patients diagnosed and treated for malaria. Two physicians independently reviewed patient folders according to the stated diagnoses. Malaria prescriptions were described as inappropriate when they do not adhere to the standard treatment guidelines. The economic cost was mainly treatment cost which was sourced as medication cost. Total and average costs for country were calculated using sample estimates and the total number of uncomplicated malaria cases that received inappropriate prescriptions.

**Results:**

The study revealed that patients received an average of two prescriptions per malaria episode. Artemether-lumefantrine (AL) was the major malaria medication (79.5%) prescribed to patients. Other medications usually antibiotics and vitamins and minerals were included in the prescription. More than 50% of prescribers did not follow the guidelines for prescribing medications to clients. By facility type, inappropriate prescription was high in the CHPS compounds (59.1%) and by ownership, government (58.3%), private (57.5%) and mission facilities (50.7%). Thus, about 55% of malaria prescriptions were evaluated as inappropriate during the review period, which translates into economic cost of approximately US$4.52 million for the entire country in 2016. The total cost of inappropriate prescription within the study sample was estimated at US$1,088.42 while the average cost was US$1.20.

**Conclusion:**

Inappropriate prescription for malaria is a major threat to malaria management in Ghana. It presents a huge economic burden to the health system. Training and strict enforcement of prescribers’ adherence to the standard treatment guideline is highly recommended.

## Background

In Ghana, malaria remains a major cause of morbidity and mortality among the population. Between 2009 and 2016, it was reported that malaria contributed between 31% and 44% of outpatient department cases [1–6]. To date, it remains one of the leading health challenges in the country; with an estimated 2.3 million suspected malaria cases needing treatment in the first quarter of 2017 alone [7].

The WHO has over the decade encouraged the Test, Treat and Track Policy (T3 Policy) for the management of uncomplicated malaria cases. The T3 policy outlines that febrile patients must be tested for malaria with a rapid diagnostic test kit (RDT) or by microscopy before treatment. Additionally, the policy also encourages the screening of febrile patients for other non-malarial illnesses [8]. The inappropriate use of medicines remains a major global issue facing many health systems [9]. The inappropriate use of antimalarial medicines by clinicians, general medical practitioners, and health facilities is most common in many developing countries [10]. This is mostly due to the weak health systems, where mechanisms for routine monitoring and supervision of medicine use are not well developed. Such inappropriate prescription not only raises concerns about resistance to anti-malarial, but also has cost implications for management of the disease, burdening further financing schemes. Thus, efficiency in the prescription of anti-malarial could free up resources for use in the control of the disease.

Studies on inappropriate prescriptions for malaria have reported varied proportions of malaria prescriptions inappropriately done, ranging from 12% to 76.4% in Nigeria and Ghana respectively among children [11, 12] to approximately 74% in India [13]. Other studies report 54.7% in Nigeria [14] and 40% in Sudan [15]. Further, Aina [14] and Uzochukwu [16] both in Nigeria report the average economic cost of inappropriately prescribed malaria medicines to be US$1.03 and US$1.70, respectively. While there are several studies reporting inappropriate prescriptions for malaria, a few studies have reported the economic cost of inappropriate prescriptions for malaria in Africa [14, 16].

This study thus set out to estimate the institutional costs associated with inappropriate prescription for uncomplicated malaria in Ghana. This study provides evidence of the economic burden of inappropriate prescription on health systems; and how effective implementation and adherence to Standard Treatment Guidelines (STG) will bring about cost savings.

## Methods

### Study setting

In Ghana, the Ghana Health Service is the major institution responsible for service delivery in all government facilities. Mission facilities are the second largest provider of health care services within the country, followed by private health care providers. At the district level, curative services are provided by district hospitals. Public health units of the district hospitals are also responsible for preventive health service. At the sub-district level, both preventive and curative services are provided by the health centres, as well as outreach services to the communities within their catchment areas. Basic preventive and curative services for minor ailments are addressed at the community and household level through the Community-based Health Planning and Services (CHPS). The CHPS compounds are the most basic level of community-based health care provision. In many lower levels of healthcare, physician assistants are among the main providers of diagnosis and treatment. In Ghana, physician assistants are healthcare professionals trained in the medical model to practice medicine. Every level of the healthcare services usually includes malaria care. The role of clinicians and the various health facilities in terms of ownership and level matters in the management of malaria including cost of inappropriate prescription.

### Study design and aim

This study design was cross-sectional, reviewing patient folders at one-time point, and using the cost-of-illness approach to estimate the value of resources expended on malaria treatments outside the STG, which could have been saved.

### Sampling of facilities and folders

The country was stratified into three ecological zones to ensure a representation of the diversity of the issues. These were: (1) Northern – Upper East, Upper West and, Northern; (2) Central – Brong Ahafo and Ashanti; and (3) Southern – Volta, Eastern, Greater Accra, Central and Western. One region each was selected to represent one zone. A list of districts within each region was obtained from the Centre for Health and Information Management/Policy Planning, Monitoring, and Evaluation Division of Ghana Health Service (CHIMS/PPME—GHS). Two districts were randomly selected in each region (one rural, one urban). In each district, the different levels and ownership of facilities were considered, namely public, mission and private. A total of 27 facilities were selected for this study (Table 1).

**Table 1:** Number of folders sampled per facility

Region	District	Facility	Facility ownership	Number of folders	
Volta	North Tongu	Health facility 1	CHAG	141	
		Health facility 2	Government	40	
		Health facility 3	Government	30	
		Health facility 4	Private	30	
	Keta Municipal	Health facility 1	Government	106	
		Health facility 2	CHAG	42	
		Health facility 3	Government	74	
		Health facility 4	Private	30	
		Health facility 5	Government	30	
Upper East	Bawku	Health facility 1	CHAG	142	
		Health facility 2	Private	128	
		Health facility 3	Government	30	
		Health facility 4	Government	31	
		Health facility 5	Government	30	
	Kasena Nankana	Health facility 1	Government	96	
		Health facility 2	CHAG	45	
		Health facility 3	Government	30	
		Health facility 4	Government	30	
Brong-Ahafo	Wenchi	Health facility 1	CHAG	147	
		Health facility 2	Private	87	
		Health facility 3	CHAG	55	
		Health facility 4	Public/Government	50	
		Health facility 5	Public/Government	30	
	Nkoranza South	Health facility 1	CHAG	54	
		Health facility 2	Private	49	
		Health facility 3	Public/government	38	
		Health facility 4	Public/government	30	
Total				1625	

#### Sample size for patient folders for all regions

This study was designed primarily to estimate the mean cost of inappropriate prescriptions in health facilities. Assuming that the cost follows the Gaussian distribution, the equation used in estimating the required sample size is given as follows:

$$n=\frac{{\sigma }^{2}{\left({Z}_{\frac{\alpha }{2}}\right)}^{2}(1+f)}{{e}^{2}}Design\,effect$$

Where ${Z}_{\frac{\alpha }{2}}=1.96$ is the standard normal deviation corresponding to 95% significance criterion, $f=10.0\%$ is the non-response rate, $e$= 0.05 is the margin of error and        $\sigma =0.75$ is the standard deviation of cost from 2015 National Health Insurance Scheme tariff.

Since $\sigma$ has not been estimated from previous studies in Ghana, we approximated it using

$$\sigma \, = \,{\raise0.7ex\hbox{$r$} \!\mathord{\left/ {\vphantom {r 4}}\right.\kern-\nulldelimiterspace} \!\lower0.7ex\hbox{$4$}}$$

where r is the range of cost from public primary care hospitals (GHS7.20–10.20). Assuming a design effect of 1.709, the estimated sample size for the study was 1625 patients. This sample size was then allocated to regions (first) and facilities based on the population of patients proportional to size.

Once the total sample for each facility for the year was estimated, simple random sampling was used to allocate the sample across the months (since malaria OPD attendance was not uniform across months). This was done by first obtaining the OPD attendance register for the year 2016. The sampling frame was OPD malaria attendance for the year 2016. Secondly, the monthly breakdown of OPD malaria cases for the year 2016 was obtained from the register, and identification numbers for folders of malaria patients were recorded. Then, the sample for the month was calculated based on the formula:

$$\left( {\frac{A}{B}} \right) \times C$$

where, A = malaria cases for month y in each facility. B = total malaria cases for the year 2016 in each facility. C = total sample size calculated for each facility.

The calculation was done for all the months (n = 12) in the year 2016. Thirdly, after obtaining the sample size distribution by month for 2016, all the patients’ folder numbers for each month were listed and the sample was drawn randomly. The process was repeated for all 12 months of the year 2016.

##### Inclusion criteria

Patients’ folders containing a diagnosis of malaria, and explicitly specified as such in the folder.

##### Exclusion criteria

Patients recorded to have malaria in OPD register, but malaria diagnosis not clearly written or missing in patient’s folder.

### Data extraction and variables

A data extraction form was used to gather patient prescription information from their folders. Data from the folders were collected by trained research assistants guided by prescribers in each facility. Using the STG for uncomplicated malaria, prescriptions were assessed and classified into inappropriate and appropriate prescriptions. Prescriptions that were not supported by the diagnosis of uncomplicated malaria or any of the other recorded, additional diagnoses were deemed to be inappropriate. Variables recorded include data on age, sex, diagnoses, the anti-malarial medicines prescribed to the patients and other medicines prescribed for the additional, stated diagnoses.

### Data analysis

The average number of medicines per encounter was estimated. This was calculated as: Average number of medicines prescribed per encounter (C) = Total number of medicines prescribed (B) / number of encounters surveyed (A). The analysis also took into consideration the doses and formulation in the analysis. Further analysis of the data provided additional indices such as the pattern of prescription by Standard Treatment Guideline [17] among prescribers, frequency of anti-malarial prescriptions by the level of facility and, prescriptions by therapeutic groups, including antibiotic prescription, analgesic prescription pattern, anti-malarials. Finally, two physicians, using the STG as the specified criteria, independently assessed all prescription patterns with malaria diagnosis and other recorded diagnoses, where recorded, this is used to determine whether the guide was followed, and the prescription was rational. Disagreements were resolved by discussion and, if necessary, a third independent person was involved.

Primarily, the STG for Malaria in Ghana recommends artemisinin-based combination therapy (ACT) for pharmacological treatment with 1st Line Treatment as Evidence Rating: [A] Artesunate + Amodiaquine, oral, or Artemether + Lumefantrine, oral, or Dihydroartemisinin + Piperaquine, oral.

### Determination and definition of inappropriate malaria prescriptions

Appropriate prescriptions were determined as those based on appropriate examinations to test for malaria, diagnosis and then prescription of medicines per the recommendations in the STG. Other prescribed medication was verified against other additional stated diagnoses for a decision on appropriateness. If prescription did not follow the STG recommendation, and if there was no additional, stated diagnoses that would explain the extra medicines, then the prescription was deemed inappropriate.

### Determination of the proportion of inappropriate malaria prescriptions

Based on the records samples per facility type, the proportion of inappropriate prescriptions (i.e., not adhering to STG and not supported by additional, stated diagnoses) was estimated from the total sample and expressed as the percentage of prescriptions for all uncomplicated malaria cases. A national estimate was calculated based on the sample estimates, number of regions, number of districts and number and type of health facilities in the country.

### Estimation of cost of inappropriate prescription (institutional cost)

The institutional cost of inappropriate prescriptions for uncomplicated malaria comprised medication cost. The cost of inappropriate prescription included the cost of medications prescribed outside the STG. The costs were determined using prices obtained from the Public Procurement Authority of Ghana (PPA) and average cost estimates from the local market. Total and average costs were calculated. Results of all cost were reported in the United States (US) dollar using Bank of Ghana annual average interbank exchange rate, 2016 (US$1.00 equivalent to GHS4.30).

### Ethical approval

As a programmatic review for the National Malaria Elimination Programme, permission for data collection was obtained from the Ghana Health Service Headquarters and Christian Health Association of Ghana, Regional and District Directors, and facility heads.

## Results

### Characteristics of patients in folders

About 1625 folders of patients diagnosed with malaria were reviewed across the three regions. The analysis revealed that about 62% (n = 1005) of patients whose folders were reviewed were females. Moreover, about a fifth of the folders was for children < 5 years (22.2%) and 16% were above 50 years. Approximately 73% (n = 1189) of patients diagnosed with malaria tested positive, 4.5% (n = 73) tested negative while 22.3% (n = 363) were not tested (Table 2).

**Table 2:** Characteristics of patients in folders

	Upper east	Volta	Brong Ahafo	Total
	n (%)	n (%)	n (%)	n (%)
Age				
0–4	123 (21.3)	64 (12.6)	173 (32.0)	360 (22.2)
5–9	90 (15.6)	64 (12.6)	97 (18.0)	251 (15.4)
10–19	118 (20.4)	94 (18.5)	77 (14.3)	289 (17.8)
20–29	72 (12.5)	58 (11.5)	52 (9.6)	182 (11.2)
30–39	43 (7.4)	62 (12.2)	31 (5.7)	136 (8.4)
40–49	41 (7.1)	65 (12.8)	40 (7.4)	146 (9.0)
50+	91 (15.7)	100 (19.7)	70 (13.0)	261 (16.1)
Total	578 (100)	507 (100.0)	540 (100.0)	1625 (100)
Sex				
Female	347 (60.0)	336 (66.3)	322 (59.6)	1005 (61.8)
Male	231 (40.0)	171 (33.7)	218 (40.4)	620 (38.2)
Total	578 (100.0)	507 (100.0)	540 (100.0)	1625 (100.0)
Test results				
Positive	555 (96.0)	300 (59.2)	334 (61.9)	1189 (73.2)
Negative	14 (2.4)	36 (7.1)	23 (4.3)	73 (4.5)
Not tested	9 (1.6)	171 (33.7)	183 (33.9)	363 (22.3)
Total	578 (100.0)	507 (100.0)	540 (100.0)	1625 (100.0)

### Prescription patterns (decisions on prescription pattern)

The analysis revealed that overall, about 44% of malaria prescriptions were appropriately done while 55% were inappropriate. In terms of prescription pattern by prescriber type, it was found that nurses were the least compliant to the STG, with about six (6) out of every ten (10) malaria prescriptions (61%) given by nurses being inappropriate (Table 3). Similarly, medical officers were less compliant with the STG with more than one in every two malaria prescriptions being inappropriate. Physician assistants were the only group among prescribers that recorded less than half of inappropriate prescription (47.6%). Again, the analysis by facility type showed that the inappropriate prescription across all facilities was more than half, ranging from 52.8 to 59.1%. CHPS compounds recorded the highest level of non-compliance to the STG (59.1%). In addition, the facility ownership showed significant non-compliance to the STG, with health facilities owned by the government recording 58.3% of inappropriate prescriptions. This was followed by private health facilities (57.5%) and mission health facilities (50.7%). Further, across all age categories in this study, inappropriate prescriptions were more than 50% except for prescriptions among children < 5 years (48.9%) (Table 3).

**Table 3:** General pattern of malaria prescription and selected variables

Description	Appropriate	Inappropriate	Undecided	Total	Chi-square
Malaria test result	n (%)	n (%)	n (%)	n (%)	
Positive	694 (58.4)	483 (40.6)	12 (1.0)	1189 (100)	**0.000**
Negative	7 (9.6)	63 (86.3)	3 (4.1)	73 (100)	
Not tested	9 (2.5)	353 (97.2)	1 (0.3)	363 (100)	
Age group					**0.056**
<5	180 (50.0)	176 (48.9)	4 (1.1)	360 (100)	
5–12	151 (41.8)	208 (57.6)	2 (0.6)	361 (100)	
13–17	66 (48.2)	70 (51.1)	1 (0.7)	137 (100)	
18–59	237 (40.0)	347 (58.5)	9 (1.5)	593 (100)	
Above 60	76 (43.7)	98 (56.3)	0 (0.0)	174 (100)	
Sex					**0.208**
Male	263 (42.4)	348 (56.1)	9 (1.5)	620 (100)	
Female	447 (44.5)	551 (54.8)	7 (0.7)	1005 (100)	
Facility type					**0.084**
CHPS compound	67 (38.1)	104 (59.1)	5 (2.8)	176 (100)	
Health centre	151 (46.6)	171 (52.8)	2 (0.6)	324 (100)	
Private clinic/hospital	173 (43.4)	221 (55.4)	5 (1.3)	399 (100)	
Hospital	319 (43.9)	403 (55.5)	4 (0.6)	726 (100)	
Facility ownership					**0.039**
Mission	292 (48.7)	304 (50.7)	4 (0.7)	600 (100)	
Private	148 (41.3)	206 (57.5)	4 (1.1)	358 (100)	
Public	270 (40.5)	389 (58.3)	8 (1.2)	667 (100)	
Type of prescriber					**0.000**
Medical officer	433 (43.2)	561 (56.0)	8 (0.8)	1002 (100)	
Physician assistant	163 (51.4)	151 (47.6)	3 (0.9)	317 (100)	
Nurse (CHN, EN etc.)	114 (37.3)	187 (61.1)	5 (1.6)	306 (100)	
Total	710 (43.6)	899 (55.3)	16 (1.0)	1625 (100)	

### Pattern of artemisinin-based combination prescribed

**Fig. 1:**
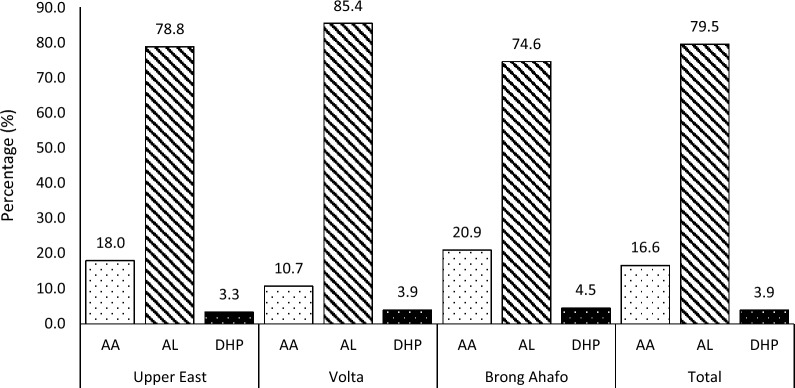
Proportion of treatments ACTs

Figure 1 shows the proportion of malaria treatment using ACT as recommended by the World Health Organization (WHO). The commonest treatment combination was artemether- lumefantrine (AL), accounting for approximately 4 out of every 5 artemisinin-based combinations prescribed (79.5%), followed by artesunate-amodiaquine, AA (16.6%) and dihydroartemisinin-piperaquine, (DHP), which is about 3.9%.

### Number and cost of other medications prescribed by therapeutic categories

Table 4 shows other medications given to patients and their associated costs. These medications totalled $1088.42 per year for the sample. The majority of this cost was due to additional malaria medications other than artemisinin-based combinations ($384.21). Vitamins and mineral combinations formed the majority (33.5%) of other medications given, followed by additional non-ACT anti-malarials (14.7%), antibiotics (12.2%) and haematinics (9.2%). The average cost of these additional medications ranged between US$0.49 and US$3.72 (Table 4).

**Table 4:** Number and cost of other medications prescribed by therapeutic categories

Therapeutic group	n (%)	Total cost US$	Average cost US$
Analgaesic/antipyretics	37 (5.3)	37.72	1.02
Antibiotics	85 (12.2)	149.80	1.76
Antidiabetics	14 (2)	52.02	3.72
Antifungal	12 (1.7)	11.80	0.98
Antihelmintics	42 (6)	82.96	1.97
Antihistamines	14 (2)	7.53	0.54
Antihypertensives	21 (3)	44.19	2.10
Additional antimalarial (non-ACT)	102 (14.7)	384.21	3.77
Cough expectorant/antitussives	19 (2.7)	24.59	1.30
Haematinics	64 (9.2)	70.09	1.10
Vitamin and mineral combinations	233 (33.5)	114.24	0.49
Glucose-elevating agents	22 (3.2)	51.16	2.33
Other*	31 (4.5)	58.09	1.87
Total	696 (100)	1088.42	1.56

* Antacids & Laxatives, Anti-asthmatics, Anticonvulsants, Antidepressants, Nonsteroidal Anti-inflammatory Medicines, Proton Pump Inhibitor, Sterile irrigating solutions

Table 5 shows the average number of medicines prescribed per malaria episode and this was around 2.2 for all three regions under study. Facilities in Brong Ahafo prescribed the least number of medicines while facilities in the Volta Region prescribed the most. With regard to excess medicines prescribed, again Brong Ahafo prescribed the least while the other two regions prescribed about the same number of excess medicines (i.e., 1.2).

**Table 5:** Average number of medicines prescribed per malaria episode

Item	Upper East	Volta	Brong Ahafo	All regions
Average number of medicines prescribed per malaria episode	2.1	2.7	1.8	2.2
Average number of excess medicines prescribed per malaria episode	1.2	1.2	1.1	1.2

### Cost of prescription outside STG

The total cost (average cost) of inappropriate prescription for the study sample was estimated at US$1,088.42 (US$1.20) which is mainly the cost of medication. The regional estimates of the cost of inappropriate prescription were extrapolated to national estimates using total OPD cases for uncomplicated malaria for 2016. The total number of OPD cases for uncomplicated malaria was 6,812,045 and given that the percentage of inappropriate prescription was 55.3%, this translates to 3,767,066 cases for which prescriptions were inappropriate. Also, given that the average cost was US$1.20, the total cost of inappropriate prescription at the national level was estimated to be US$4,520,479.20 (Table 6).

**Table 6:** Cost of inappropriate prescription (i.e., outside STG)

Cost component	Upper east	Volta	Brong Ahafo	Total cost	Average cost
Medication	315.09	590.43	182.90	1,088.42	1.20
**Total cost**	315.09	590.43	182.90	1,088.42	1.20

The regional estimates of the cost of inappropriate prescription were extrapolated to national estimates using total OPD cases for uncomplicated malaria for 2016. The total number of OPD cases for uncomplicated malaria was 6,812,045 and given that the percentage of inappropriate prescription was 55.3%, this translates to 3,767,066 cases for which prescriptions were inappropriate. Also, given that the average cost was US$1.20, the total cost of inappropriate prescription at the national level was estimated to be US$4,520,479.20.

## Discussion

The study revealed that about 55% of malaria prescriptions did not follow the standard treatment guidelines, translating into approximately 3.7 million malaria prescriptions (in 2016) that did not follow the STG, nationally. The study has estimated the economic cost of inappropriate prescriptions for uncomplicated malaria in Ghana to be approximately US$4.52 million.

The finding that 55% of malaria prescriptions in this study were inappropriately done is similar to the finding of 54.7% reported in Nigeria [14]; significantly lower than the 73.9% reported in India [13], and higher than the 40% and 32% reported in Sudan and India, respectively [15] and 12% in Nigeria [11]. It is not immediately clear what factors account for the marked differences between the current study’s estimate (55%) and those of Mishra et al. [13] and Ishola et al. [11] – i.e., about 74% and 12%, respectively. However, it is important to note that those two studies focused on malaria in children, while the current study focuses on malaria in the general population. Additionally, the current study’s finding that the average number of medicines prescribed per malaria episode was 2.2 is lower than those reported elsewhere in the sub-region i.e., 6.8 by Uzochukwu et al. [16] and 5.4 by Ishola et al. [11], both in Nigeria.

The current study estimates that the average economic cost of inappropriate malaria prescriptions in Ghana was US$2.77, which is relatively higher than US$1.03 [14] and US$1.70 [16] reported in Nigeria. However, the differences in data year and economic context may have contributed to the differences, aside from other factors. Again, this study has estimated the economic cost of non-adherence of malaria prescriptions to STG in Ghana to be approximately US$10.4 million for the year 2016. This cost could have translated into savings for the National Health Insurance Scheme (in terms of saved reimbursements), government (in terms of saved staff time, supplies and other overheads), individuals and households (in terms of saved out-of-pocket expenditures). Although this current study focused on the direct cost of inappropriate malaria prescription, it is important to emphasize that, there could be potential additional cost to the health system due to misdiagnoses as having malaria. As seen in previous studies, these misdiagnoses are costly financially, and the result in potential treatment delays which may put patients at risk [18–20].

The findings of this study are useful to the health sector of Ghana in several ways. First, it is important for the NHIA to intensify claims vetting to minimize losses accruing to the NHIS/government due to inappropriate malaria prescriptions. Second, the estimates on the cost of inappropriate malaria prescriptions provide an indication of the economic burden of inappropriate prescriptions for malaria to the health system indicating where interventions are needed to target prescriber behaviour. There is the need for NMEP and other stakeholders to intensify training programmes targeting all prescribers while engaging them on the STGs, in order to reduce the inappropriate malaria prescriptions.

In this work, the limitation in the analysis is acknowledged. Foremost, further analysis considering co-morbidities that were not recorded in the patient’s folder may have provided additional value to the study, however, this was not the main focus of the study and data on this was not readily available. Moreover, the study did not disaggregate the cost in a sub-analysis which considered those cost covered out-of-pocket by patients and cost covered by the NHIS, slide negative versus slide positive, and male versus female. Future studies may consider these sub-analyses. In addition, given the time that has elapsed, it is recommended that future studies will aim at evaluating the interventions implemented by the NMEP to address the problem of inappropriate prescription in Ghana.

### Policy implications

Following the study findings, the National Malaria Elimination Programme (NMEP) in its bid to reduce drastically inappropriate prescription in Ghana organized case management training for both private and public health facilities biennially with behaviour change modules incorporated in the training modules. This group training is interspersed with on-site training and supportive supervision in the facilities. During these visits, all thematic areas are assessed (consulting room, laboratory, pharmacy) and action plans are developed with facility managers to help track the resolution of identified challenges. Annual peer review meetings for facilities at the same level to learn from each other have been started. The programme has also put together a team that follows up quarterly on facilities that report any form of non-compliance to the treatment protocols to help promptly deal with cases of inappropriate prescription. Facilities also use customized approaches to address the issues of inappropriate malaria prescription, including pharmacies instructed not to serve unconfirmed malaria cases with malaria medicines as well as discussion of inappropriate prescription issues in ward rounds and clinical meetings when they come up. Additionally, together with other stakeholders, stock level monitoring across all facilities is being scaled up through the Ghana Integrated Logistics Management and Information System, which is a national logistics management system. This is to help reduce the issues with logistic stock out which could also influence the appropriateness of prescription.

## Conclusion

The study has estimated the economic cost of inappropriate malaria prescriptions in Ghana to be approximately US$4.52 million for 2016, providing the economic burden that inappropriate prescribing behaviour presents to the health system. It is a good background for the National Health Insurance Authority and Ministry of Health to target for cost containment purposes and improved financing. Moreover, inappropriate prescribing is a threat to the quality of life of patients and a potential menace for the anti-malarial drug resistance development. It is crucial for the NMEP to sustain intervention that followed the study to prevent the future development of anti-malarial drug resistance.

## Data Availability

The datasets analysed during the current study are not publicly available due because there is institutional control but are available from the corresponding author on reasonable request.

## Abbreviations

ACT: Artemisinin-based combined therapy
CHAG: Christian Health Association of Ghana
CHPS: Community-based health and planning services
IPTp: Intermittent preventive treatment in pregnancy
IRS: Indoor residual spraying
ITN: Insecticide-treated mosquito net
MOH: Ministry of Health
NMEP: National malaria elimination programme
NHIA: National health insurance authority
NHIS: National health insurance scheme
RDT: Rapid diagnostic test
SMC: Seasonal malaria chemotherapy
SSA: Sub-Saharan Africa
STG: Standard treatment guideline
WHO: World Health Organization
